# Supplementary material for: Extrinsic MMPs drive epithelial shape change via basal extracellular matrix disassembly in the Drosophila wing disc
Source: Commun Biol. 2026 May 29;9:1447. doi: 10.1038/s42003-026-10376-9 (PMC13728232; doi:10.1038/s42003-026-10376-9)
Supplement: Supplementary file 1 [file 42003_2026_10376_MOESM1_ESM.pdf]

Supporting information

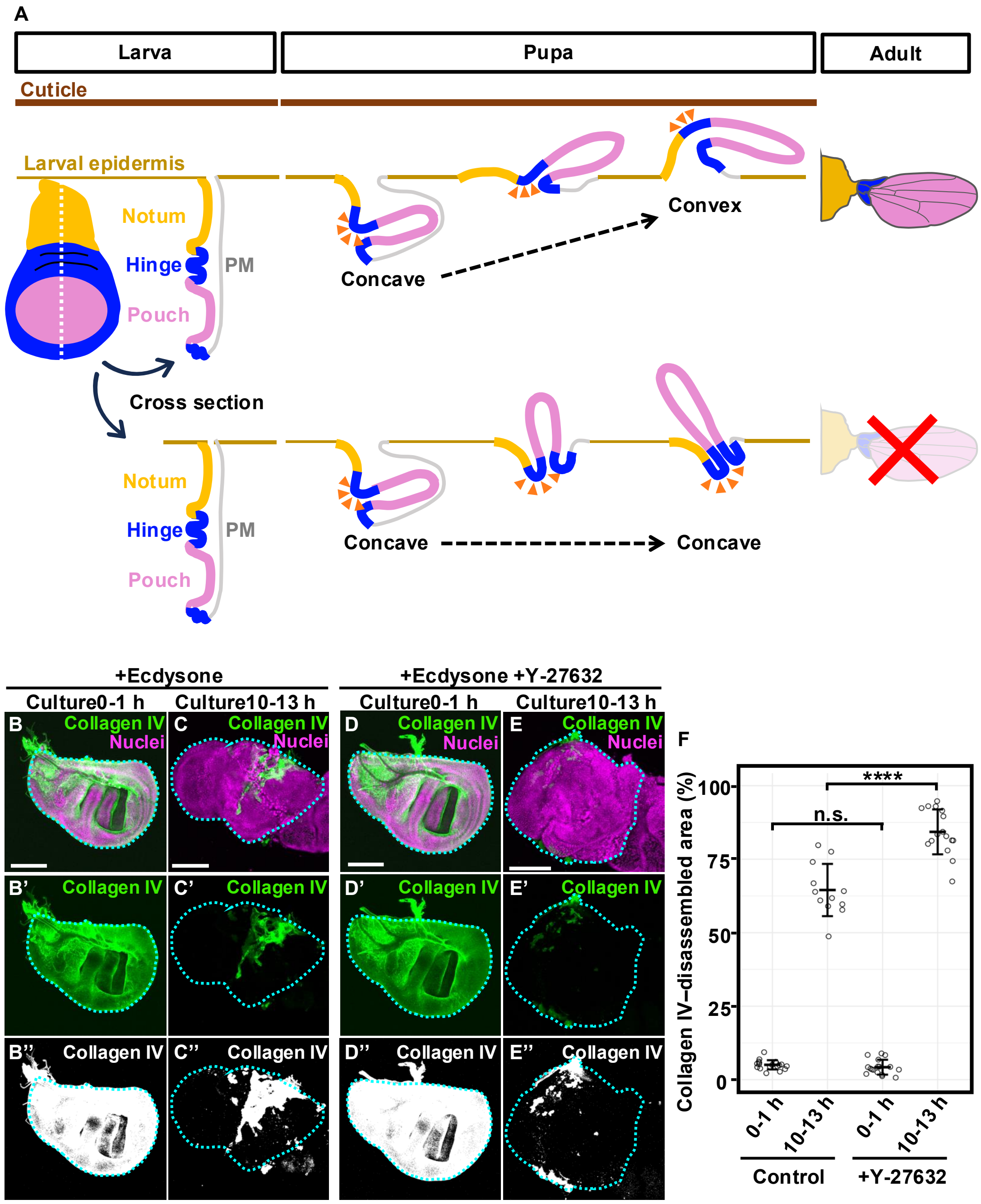

S1 Fig, related to Figure 1. The wing disc undergoes a concave-to-convex shape transition during the larval-to-pupal stage

(A) Schematic diagrams illustrate the *in vivo* morphological transition of the wing disc during the larval-to-pupal stage. Orange arrowheads indicate the same anatomical position before and after the curvature change, marking the region that undergoes the

concave-to-convex transition. Upper panels illustrate the concave-to-convex transition of the epithelium; lower panels show the epithelium remaining concave.

(B–E'') Wing discs carrying *viking::GFP* (green) and *ubi-RFP* (magenta) were dissected and treated with ecdysone for 0–1 h or 10–13 h in the presence of either DW (B–C'') or the ROCK inhibitor Y-27632 (D–E''). The black-and-white images (B''), (C''), (D''), and (E'') represent the binarized versions of (B'), (C'), (D'), and (E'), respectively. Scale bar, 100 μm.

(F) Dot plots showing the percentage of Collagen IV disassembled area on the basal side, with horizontal bars indicating the mean ± SD, corresponding to the representative images shown in (B'') (n = 19), (C'') (n = 12), (D'') (n = 17), and (E'') (n = 16). ****p<0.0001, n.s., not significant; Wilcoxon rank-sum test.

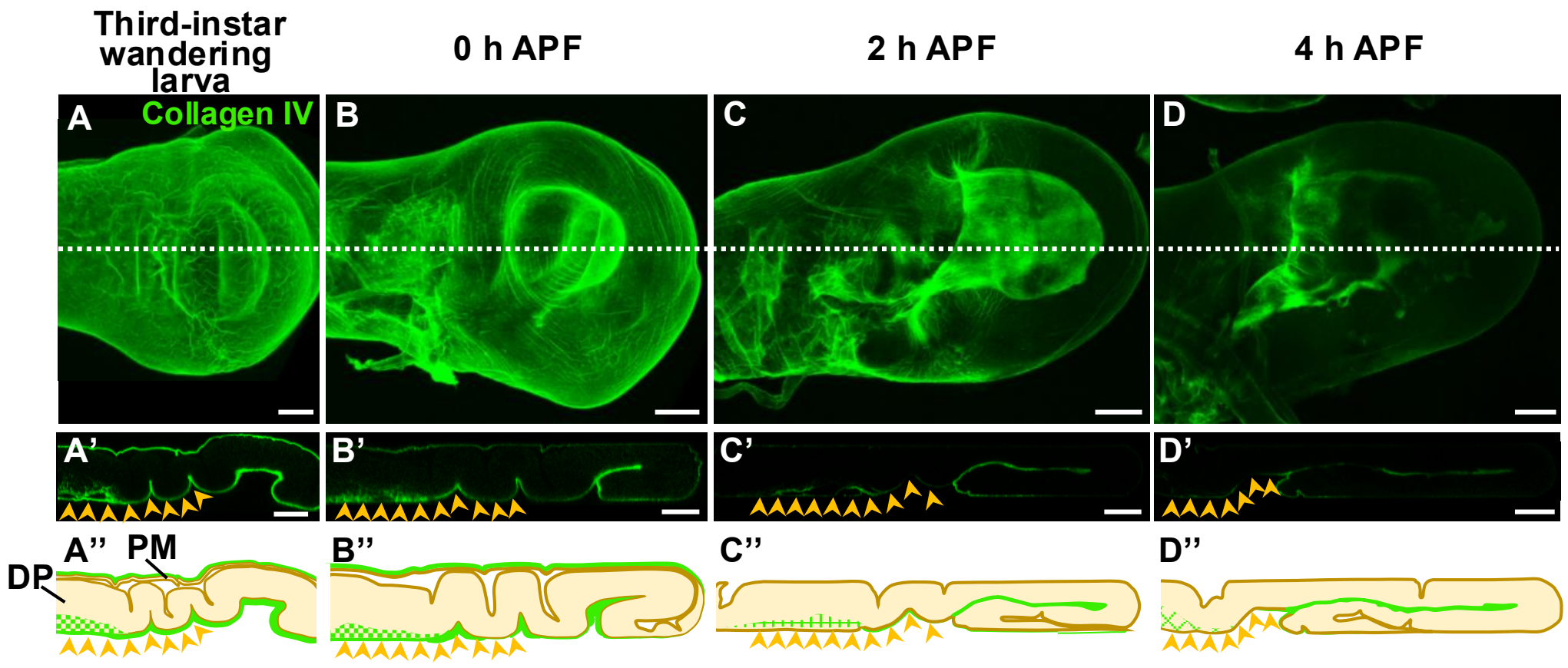

S2 Fig, related to Figure 2. MMP-dependent disassembly of the basal ECM is required for the epithelial concave-to-convex shape transition

(A–D') Wing discs carrying *viking::GFP* (green) were dissected at the third-instar wandering larval stage (A), the white pupal stage (0 h APF) (B), 2 h APF (C), or 4 h APF (D). Corresponding x–z cross-sections, indicated by white dotted lines, are shown in (A', B', C', and D'). (A'')–(D'') show schematic representations of these x–z cross-sections. Orange arrowheads indicate the basal regions of the notum and hinge where Collagen IV undergoes progressive disassembly, with signal intensity gradually decreasing and becoming barely detectable by 4 h APF. PM, peripodial membrane; DP, disc proper. Scale bar, 50 µm.

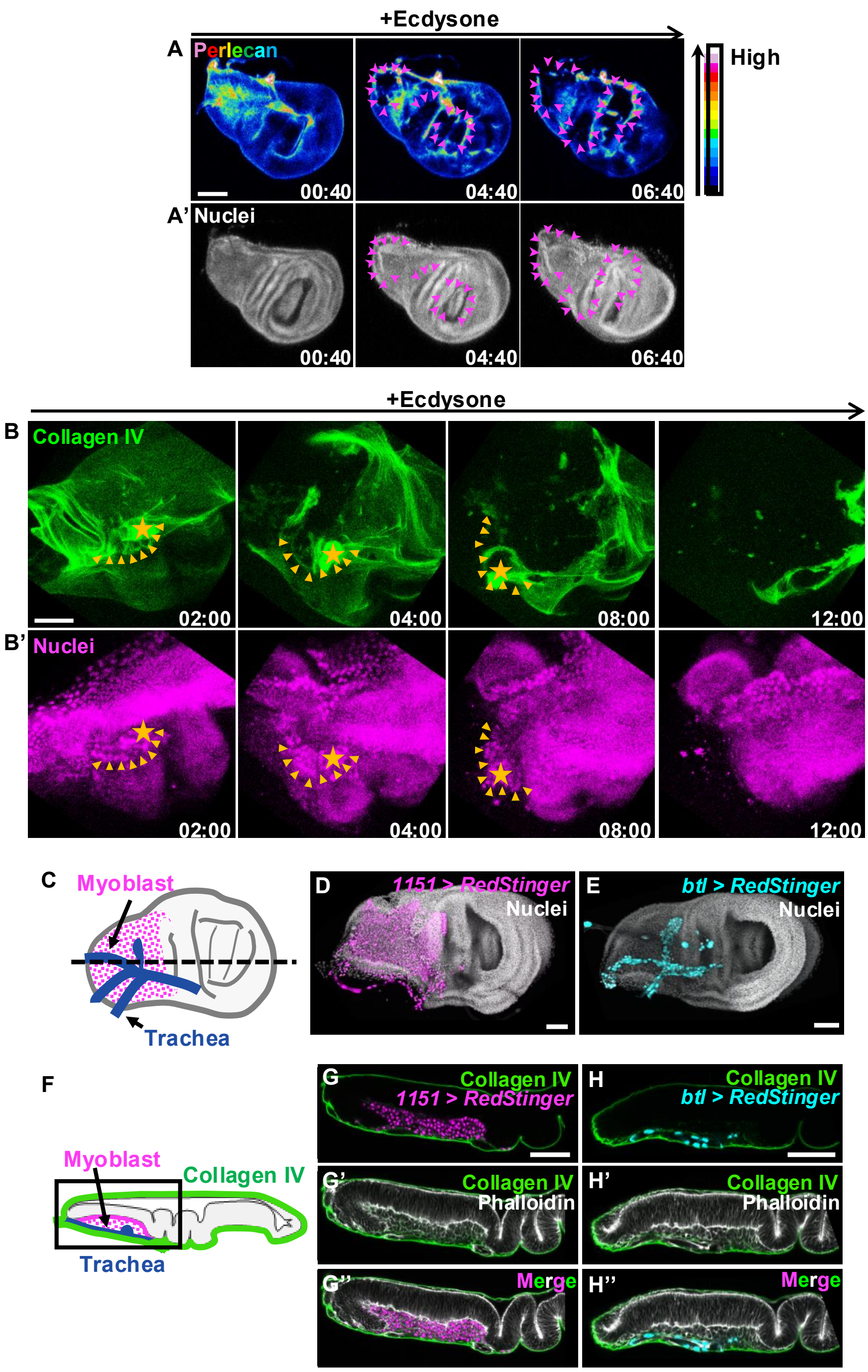

S3 Fig, related to Figure 3. Myoblasts and tracheal cells are spatially associated with localized basal ECM disassembly during the larval-to-pupal transition

(A–A') Time-lapse confocal imaging of an ecdysone-treated wing disc carrying *trol-GFP* (pseudocolor) and *ubi-RFP* (white). Magenta arrowheads indicate the regions undergoing Perlcan disassembly. Time is shown as h:min from the onset of ecdysone treatment. Scale bar, 100 µm.

(B–B') Time-lapse confocal imaging of an ecdysone-treated wing disc carrying *viking::GFP* (green) and *ubi-RFP* (magenta). Orange arrowheads and stars indicate

non-epithelial cells that migrate in association with Collagen IV disassembly. Time is shown as h:min from the onset of ecdysone treatment. Scale bar, 50 µm.

(C–E) Wing discs expressing *RedStinger* under the control of *1151-Gal4* (magenta) (D) or *btl-Gal4* (cyan) (E) were dissected at the third-instar wandering larval stage, stained with DAPI (white), and imaged from the basal side using a Zeiss LSM900 confocal microscope. Schematic diagram (C) illustrates the representative image shown in (D) and (E). Scale bar, 50 µm.

(F–H'') Wing discs carrying *viking::GFP* (green) and expressing *RedStinger* under the control of *1151-Gal4* (magenta) (G–G'') or *btl-Gal4* (cyan) (H–H'') were dissected at the third-instar wandering larval stage, stained with anti-GFP antibody and Phalloidin (white), and imaged in cross-section using a Zeiss LSM900 confocal microscope at the position indicated by the black dotted line in (C). The schematic diagram in (F) illustrates the representative cross-sectional views shown in (G–G'') and (H–H''). Scale bar, 50 µm.

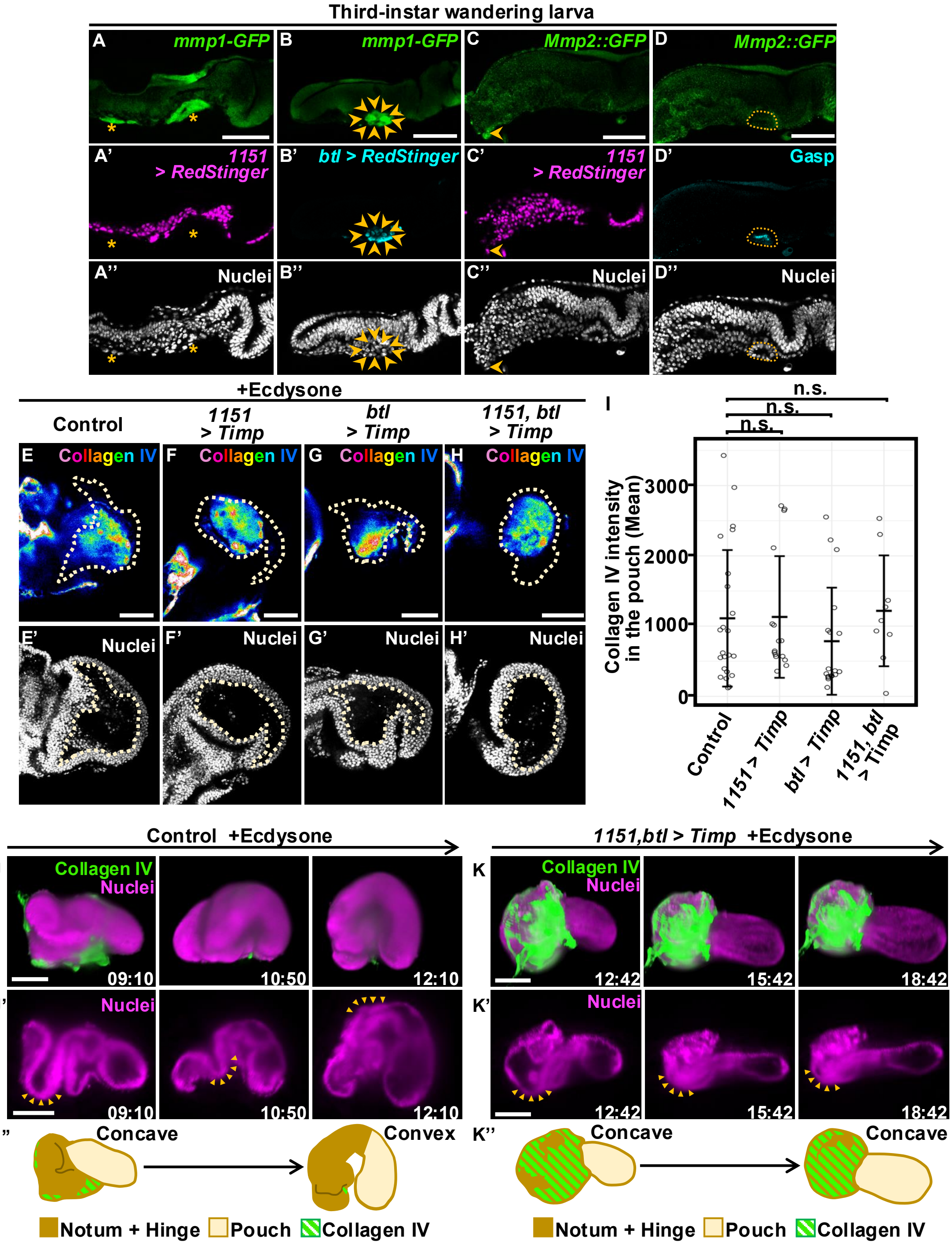

Third-instar wandering larva
A mmp1-GFP
B mmp1-GFP
C Mmp2::GFP
D Mmp2::GFP
A' 1151 > RedStinger
B' btl > RedStinger
C' 1151 > RedStinger
D' Gasp
A'' Nuclei
B''
C'' Nuclei
D'' Nuclei
+Ecdysone
Control
1151 > Timp
btl > Timp
1151, btl > Timp
E Collagen IV
F Collagen IV
G Collagen IV
H Collagen IV
E' Nuclei
F' Nuclei
G' Nuclei
H' Nuclei
I
n.s.
n.s.
n.s.
Collagen IV intensity in the pouch (Mean)
3000
2000
1000
0
Control
1151 > Timp
btl > Timp
1151, btl > Timp
Control +Ecdysone
1151,btl > Timp +Ecdysone
J Collagen IV Nuclei
09:10
10:50
12:10
J' Nuclei
09:10
10:50
12:10
K Collagen IV Nuclei
12:42
15:42
18:42
K' Nuclei
12:42
15:42
18:42
J'' Concave
Convex
K'' Concave
Concave
Notum + Hinge
Pouch
Collagen IV
Notum + Hinge
Pouch
Collagen IV

56

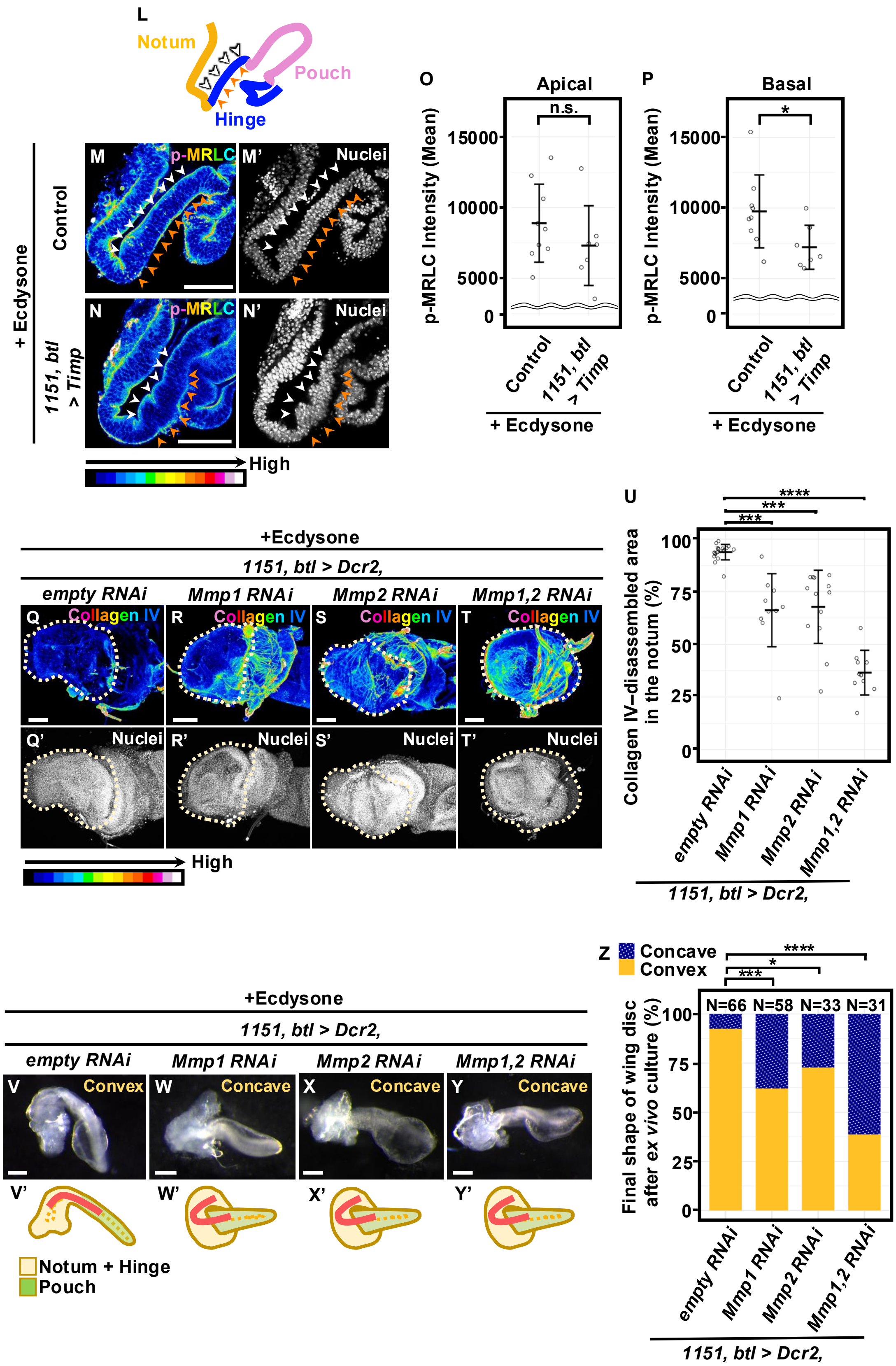

S4 Fig, related to Figure 4. Mmp1 and Mmp2 in myoblasts and tracheal cells drive Collagen IV disassembly and the epithelial shape transition

(A–B'') Wing discs carrying *mmp1-GFP* transcriptional reporter (green) and expressing *RedStinger* under the control of *1151-Gal4* (magenta) (A–A'') or *btl-Gal4* (cyan) (B–B'') were dissected at the third-instar wandering larval stage and stained with anti-GFP antibody and DAPI (white). Asterisks shown in (A) and orange arrowheads shown in (B) indicate Mmp1-positive tracheal cells. Scale bar, 50 µm.

(C–C'') A wing disc carrying the *Mmp2::GFP* knock-in reporter (green) and expressing *RedStinger* under the control of *1151-Gal4* (magenta) was dissected at the third-instar wandering larval stage and stained with anti-GFP antibody and DAPI (white). An orange arrowhead indicates an Mmp2-positive myoblast. Scale bar, 50 µm.

(D–D'') A wing disc carrying the *Mmp2::GFP* knock-in reporter (green) at the third-instar wandering larval stage was dissected and stained with anti-GFP and anti-Gasp (cyan) antibodies, together with DAPI (white). Orange dotted lines outline the tracheal tube. Scale bar, 50 µm.

(E–H') Wing discs carrying *viking::GFP* (pseudocolor) were dissected, treated with ecdysone, and subsequently stained with anti-GFP antibody and DAPI (white). Images are shown for control (E) and for discs expressing Timp under the control of *1151-Gal4* (F), *btl-Gal4* (G), or both *1151-Gal4* and *btl-Gal4* (H). Yellow dotted lines outline the pouch regions. Scale bar, 50 µm.

(I) Dot plots showing the mean Collagen IV intensity in the pouch region, with horizontal bars indicating the mean ± SD, corresponding to the representative images shown in (E) (n = 23), (F) (n = 16), (G) (n = 18), and (H) (n = 9). n.s., not significant; pairwise comparisons using the Wilcoxon rank-sum test with BH correction.

(J–K'') Time-lapse light-sheet imaging of the ecdysone-treated wing discs carrying *viking::GFP* (green) and *ubi-RFP* (magenta). Images are shown for a control disc (J–J'') and for a disc expressing Timp under the control of *1151-Gal4* and *btl-Gal4* (K–K''). (J') and (K') show x–z cross-sections corresponding to the images shown in (J) and (K). Orange arrowheads indicate the same anatomical position in the disc before and after the curvature change, marking the region that normally undergoes the concave-to-convex transition. Schematic diagrams (J'' and K'') illustrate the concave and convex shapes corresponding to the images shown in (J) and (K). Time is shown as h:min from the onset of ecdysone treatment. Scale bar, 100 µm.

(L) Schematic of the wing disc. The hinge region is shown in blue. White and orange arrowheads indicate the apical and basal sites in the left hinge region, respectively, where p-MRLC intensity was quantified.

(M–N') Wing discs were dissected, treated with ecdysone, and stained with anti–p-MRLC antibody (pseudocolor) together with DAPI (white). Representative images are shown for control discs (M, M') and for discs expressing Timp under the control of *1151-Gal4* and *btl-Gal4* (N, N'). White and orange arrowheads indicate the apical and basal sites in the left hinge region, respectively, corresponding to those shown in (L). Scale bar, 50 μm.

(O–P) Dot plots showing p-MRLC intensity at the apical and basal sites in the left hinge region indicated by arrowheads in (L–N'). Horizontal bars indicate the mean ± SD. The data correspond to the conditions shown in (M) (n = 9) and (N) (n = 7). Apical and basal data are shown in (O) and (P), respectively. *p<0.05, n.s., not significant; Wilcoxon rank-sum test.

(Q–T') Wing discs were dissected, treated with ecdysone, and stained with anti-Collagen IV antibody (pseudocolor) and DAPI (white). Images are shown for discs expressing *empty-RNAi* (VDRC60100) (Q), *Mmp1-RNAi* (NIG4859R-1) (R), *Mmp2-RNAi* (NIG1794-1R-2) (S), or a combination of *Mmp1-RNAi* (NIG4859R-1) and *Mmp2-RNAi* (NIG1794-1R-2) (T), together with Dcr2, under the control of *1151-Gal4* and *btl-Gal4*. Scale bar, 50 μm.

(U) Dot plots showing the percentage of Collagen IV-disassembled area in the notum, where Collagen IV signals could be more reliably quantified than in the hinge region, with horizontal bars indicating the mean ± SD, corresponding to the representative images shown in (Q) (n = 19), (R) (n = 10), (S) (n = 13), and (T) (n = 10). ***p<0.001, ****p<0.0001; Kruskal-Wallis test followed by Dunn's test with Bonferroni correction.

(V–Y') Wing discs were dissected and then treated with ecdysone for 24 h. Images are shown for discs expressing *empty-RNAi* (VDRC60100) (V), *Mmp1-RNAi* (NIG4859R-1) (W), *Mmp2-RNAi* (NIG1794-1R-2) (X), or a combination of *Mmp1-RNAi* (NIG4859R-1) and *Mmp2-RNAi* (NIG1794-1R-2) (Y), together with Dcr2, under the control of *1151-Gal4* and *btl-Gal4*. Schematic diagrams (V', W', X', and Y') illustrate the concave and convex morphologies corresponding to the representative images shown in (V), (W), (X), and (Y). Scale bar, 100 μm.

(Z) Quantification of the final wing disc shape, shown as the percentage of discs that remained a concave shape or transitioned to a convex shape, corresponding to the representative images shown in (V) (n = 66), (W) (n = 58), (X) (n = 33), and (Y) (n =31). *p<0.05, ***p<0.001, ****p<0.0001; Fisher's exact test with Holm correction.

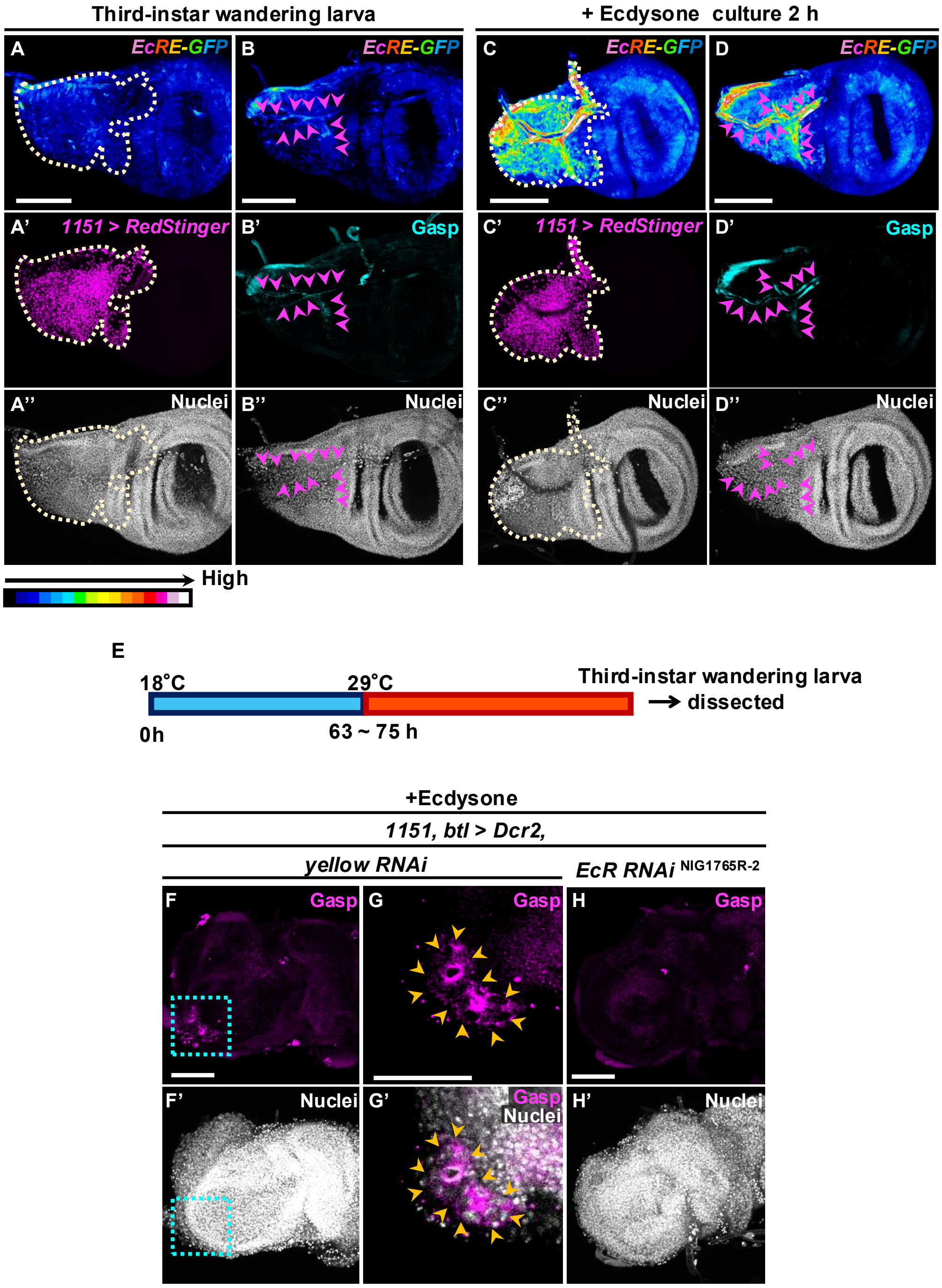

S5 Fig, related to Figure 5. Ecdysone signaling functions upstream of Mmp1 and Mmp2 to drive the epithelial shape transition

(A–D'') Wing discs carrying *EcRE-GFP* (pseudocolor) and expressing *RedStinger* under the control of *1151-Gal4* (magenta) were either dissected at the third-instar wandering larval stage (A–B'') or dissected and cultured for 2 h in the presence of ecdysone (C–D''), and stained with anti-GFP and anti-Gasp (cyan) antibodies, together with DAPI (white). Yellow dotted lines outline the myoblast population. Magenta arrowheads indicate the tracheal branches. Scale bar, 100 μm.

(E) Schematic diagrams illustrate the temperature-shift regimen used to maintain larvae carrying *Tub-Gal80*^{ts} and to induce *EcR-RNAi* expression in myoblasts and tracheal cells at specific developmental stages. Third-instar wandering larvae were subsequently dissected and cultured *ex vivo* in the presence of ecdysone prior to analysis.

(F–H') Wing discs were dissected, treated with ecdysone, and stained with anti-Gasp antibody (magenta) and DAPI (white). Images are shown for discs expressing *yellow-RNAi* (NIG3757R-1) (F–F', G–G'), or *EcR-RNAi* (NIG1765R-2) (H–H'), together with Dcr2, under the control of *1151-Gal4* and *btl-Gal4*. (G–G') show magnified views of the regions outlined by the cyan dotted lines in panels (F–F'). Orange arrowheads indicate Gasp-positive tracheal tubes. Larvae bearing *Tub-Gal80*^{ts} were maintained under the temperature conditions indicated in Fig. S5E. Scale bar, 50 µm.
