# Supplementary material for: Extrinsic MMPs drive epithelial shape change via basal extracellular matrix disassembly in the Drosophila wing disc
Source: Commun Biol. 2026 May 29;9:1447. doi: 10.1038/s42003-026-10376-9 (PMC13728232; doi:10.1038/s42003-026-10376-9)
Supplement: Supplementary file 2 [file 42003_2026_10376_MOESM2_ESM.pdf]

Description of Additional Supplementary Files

File name: Supplementary Movie 1 - Time-lapse confocal imaging of the concave-to-convex epithelial shape transition in the ecdysone-treated wing disc u sing confocal microscopy, related to Figure 1
Description: A wing disc carrying *ubi-RFP* (magenta) was dissected, cultured in the presence of ecdysone and DW as a solvent control, and subjected to time-lapse imaging at 15-min intervals using a Leica STELLARIS confocal microscope. See *Methods* for details.

File name: Supplementary Movie 2 - Time-lapse light-sheet imaging of the concave-to-convex epithelial shape transition in the ecdysone-treated wing disc, related to Figure 1
Description: A wing disc expressing *nls-SCAT3* (green) under the control of Tub-Gal4 was dissected, cultured in the presence of ecdysone, and subjected to timelapse imaging at 5-min intervals using a Zeiss Lightsheet Z.1 microscope. The left panel shows the three-dimensional shape of the wing disc, and the right panel shows the corresponding x–z cross-section. See *Methods* for details.

File name: Supplementary Movie 3 - Time-lapse confocal imaging of the ecdysone-treated wing disc reveals inhibition of the concave-to-convex epithelial shape transition by the ROCK inhibitor Y-27632, related to Figure 1
Description: A wing disc carrying *ubi-RFP* (magenta) was dissected, cultured in the presence of ecdysone and the ROCK inhibitor Y-27632, and subjected to time-lapse imaging at 15-min intervals using a Leica STELLARIS confocal microscope. See *Methods* for details.

File name: Supplementary Movie 4 - Time-lapse confocal imaging of the concave-to-convex epithelial shape transition in the ecdysone-treated wing disc under DMSO control, related to Figure 2
Description: A wing disc carrying *ubi-RFP* (magenta) was dissected, cultured in the presence of ecdysone and DMSO, and subjected to time-lapse imaging at 15-min intervals using a Leica STELLARIS confocal microscope. See *Methods* for details.

File name: Supplementary Movie 5 - Time-lapse confocal imaging of the ecdysone-treated wing disc reveals inhibition of the concave-to-convex epithelial shape transition by the MMP inhibitor GM6001, related to Figure 2
Description: A wing disc carrying *ubi-RFP* (magenta) was dissected, cultured in the presence of ecdysone and the MMP inhibitor GM6001, and subjected to time-lapse imaging at 15-min intervals using a Leica STELLARIS confocal microscope. Orange arrowheads indicate the same anatomical position in the disc, which remains concave throughout the imaging period. See *Methods* for details.

File name: Supplementary Movie 6 - Time-lapse confocal imaging of Collagen IV in an agarose-embedded, ecdysone-treated wing disc, related to Figure 3
Description: A wing disc carrying *viking::GFP* (pseudocolor) and *ubi-RFP* (white) was dissected, embedded in agarose gel, cultured in the presence of ecdysone, and subjected to time-lapse imaging at 12-min intervals using a Zeiss LSM900 confocal microscope. See *Methods* for details.

File name: Supplementary Movie 7 - Time-lapse confocal imaging of Perlecan in an agarose-embedded, ecdysone-treated wing disc, related to Figure S3
Description: A wing disc carrying *trol-GFP* (pseudocolor) and *ubi-RFP* (white) was dissected, embedded in agarose gel, cultured in the presence of ecdysone, and subjected to time-lapse imaging at 15-min intervals using a Leica STELLARIS confocal microscope. See *Methods* for details.

File name: Supplementary Movie 8 - Time-lapse confocal imaging of the migration of non-epithelial cells in association with Collagen IV disassembly in the ecdysonetreated wing disc, related to Figure S3
Description: A wing disc carrying *viking::GFP* (green) and *ubiRFP* (magenta) was dissected, mounted in an agarose gel frame, cultured in the presence of ecdysone, and subjected to time-lapse imaging at 12-min intervals using a Leica SPE confocal microscope. Orange arrowheads indicate the nonepithelial cells that migrate in association with Collagen IV disassembly. See *Methods* for details

File name: Supplementary Movie 9 - Time-lapse confocal imaging of Collagen IV and myoblast dynamics in an agarose-embedded, ecdysone-treated wing disc, related to Figure 3
Description: A wing disc carrying *viking::GFP* (green) and expressing *RedStinger* (magenta) under the control of *1151-Gal4* was dissected, embedded in agarose gel, cultured in the presence of ecdysone, and subjected to time-lapse imaging at 10-min intervals using a Leica STELLARIS confocal microscope. See *Methods* for details.

File name: Supplementary Movie 10 - Time-lapse confocal imaging of Collagen IV and tracheal cell dynamics in an agarose-embedded, ecdysone-treated wing disc, related to Figure 3
Description: A wing disc carrying *viking::GFP* (green) and expressing *RedStinger* (magenta) under the control of *btl-Gal4* was dissected, embedded in agarose gel, cultured in the presence of ecdysone, and subjected to time-lapse imaging at 10-min intervals using a Leica STELLARIS confocal microscope. See *Methods* for details.

File name: Supplementary Movie 11 - Time-lapse light-sheet imaging of the epithelial shape transition and Collagen IV disassembly in the ecdysone-treated wing disc, related to Figure S4
Description: A wing disc carrying *viking::GFP* (green) and *ubiRFP* (magenta) was dissected, cultured in the presence of ecdysone, and subjected to timelapse imaging at 20-min intervals using a Zeiss Lightsheet Z.1 microscope. The left panel shows the three-dimensional shape of the wing disc, and the right panel shows the corresponding x–z cross-section. See *Methods* for details.

File name: Supplementary Movie 12 - Time-lapse light-sheet imaging reveals that myoblast- and trachea-specific Timp expression inhibits the concave-to-convex epithelial shape transition and Collagen IV disassembly in the ecdysone-treated wing disc, related to Figure S4
Description: A wing disc carrying *viking::GFP* (green) and *ubiRFP* (magenta), and expressing *Timp* under the control of *1151-Gal4* and *btl-Gal4* was dissected, cultured in the presence of ecdysone, and subjected to time-lapse imaging at 20-min intervals using a Zeiss Lightsheet Z.1 microscope. The left panel shows the three-dimensional shape of the wing disc, and the right panel shows the corresponding x–z cross-section. See *Methods* for details.

File name: Supplementary Movie 13 - Time-lapse confocal imaging reveals that myoblast- and trachea-specific Timp expression inhibits the concave-to-convex epithelial shape transition in the ecdysone-treated wing disc, related to Figure 4
Description: A wing disc carrying *ubi-RFP* (magenta) and expressing *Timp* under the control of *1151-Gal4* and *btl-Gal4* was dissected, cultured in the presence of ecdysone, and subjected to timelapse imaging at 12-min intervals using a Leica SPE confocal microscope. See Methods for details.

File name: Supplementary Movie 14 - Time-lapse confocal imaging reveals that myoblast-specific Timp expression inhibits the concave-to-convex epithelial shape transition in the ecdysone-treated wing disc, related to Figure 4
Description: A wing disc carrying *ubi-RFP* (magenta) and expressing *Timp* under the control of *1151-Gal4* was dissected, cultured in the presence of ecdysone, and subjected to time-lapse imaging at 12-min intervals using a Leica SPE confocal microscope. See *Methods* for details.

File name: Supplementary Movie 15 - Time-lapse confocal imaging reveals that trachea-specific Timp expression inhibits the concave-to-convex epithelial shape transition in the ecdysone-treated wing disc, related to Figure 4
Description: A wing disc carrying *ubi-RFP* (magenta) and expressing *Timp* under the control of *btl-Gal4* was dissected, cultured in the presence of ecdysone, and subjected to time-lapse imaging at 15-min intervals using a Leica SP5 confocal microscope. See *Methods* for details.
