# Supplementary material for: Extrinsic MMPs drive epithelial shape change via basal extracellular matrix disassembly in the Drosophila wing disc
Source: Commun Biol. 2026 May 29;9:1447. doi: 10.1038/s42003-026-10376-9 (PMC13728232; doi:10.1038/s42003-026-10376-9)
Supplement: Supplementary file 3 [file 42003_2026_10376_MOESM4_ESM.pdf]

nature portfolio

Corresponding author(s): Chigusa Hinata, Shizue Ohsawa

Last updated by author(s): May 8, 2026

Reporting Summary

Nature Portfolio wishes to improve the reproducibility of the work that we publish. This form provides structure for consistency and transparency in reporting. For further information on Nature Portfolio policies, see our Editorial Policies and the Editorial Policy Checklist.

Statistics

For all statistical analyses, confirm that the following items are present in the figure legend, table legend, main text, or Methods section.

n/a	Confirmed	
☐	☒	The exact sample size (*n*) for each experimental group/condition, given as a discrete number and unit of measurement
☐	☒	A statement on whether measurements were taken from distinct samples or whether the same sample was measured repeatedly
☐	☒	The statistical test(s) used AND whether they are one- or two-sided<br>*Only common tests should be described solely by name; describe more complex techniques in the Methods section.*
☒	☐	A description of all covariates tested
☐	☒	A description of any assumptions or corrections, such as tests of normality and adjustment for multiple comparisons
☐	☒	A full description of the statistical parameters including central tendency (e.g. means) or other basic estimates (e.g. regression coefficient) AND variation (e.g. standard deviation) or associated estimates of uncertainty (e.g. confidence intervals)
☐	☒	For null hypothesis testing, the test statistic (e.g. *F*, *t*, *r*) with confidence intervals, effect sizes, degrees of freedom and *P* value noted<br>*Give P values as exact values whenever suitable.*
☒	☐	For Bayesian analysis, information on the choice of priors and Markov chain Monte Carlo settings
☐	☒	For hierarchical and complex designs, identification of the appropriate level for tests and full reporting of outcomes
☒	☐	Estimates of effect sizes (e.g. Cohen's *d*, Pearson's *r*), indicating how they were calculated

Our web collection on statistics for biologists contains articles on many of the points above.

Software and code

Policy information about availability of computer code

Data collection	Zeiss Zen3.4, Leica LasX(version1.7.0.147, 1.4.8.124, and 1.4.6.110), Leica AF(version: 1.0.2.71)
Data analysis	Fiji(version: 2.16.0/1.54p), Imaris x64(version:9.7.2), Rstudio(version: 2025.09.1+401), This paper does not report original code.

For manuscripts utilizing custom algorithms or software that are central to the research but not yet described in published literature, software must be made available to editors and reviewers. We strongly encourage code deposition in a community repository (e.g. GitHub). See the Nature Portfolio guidelines for submitting code & software for further information.

Data

Policy information about availability of data

All manuscripts must include a data availability statement. This statement should provide the following information, where applicable:

- Accession codes, unique identifiers, or web links for publicly available datasets
- A description of any restrictions on data availability
- For clinical datasets or third party data, please ensure that the statement adheres to our policy

All the raw data for quantitative analysis in this study have been deposited at Figshare (https://doi.org/10.6084/m9.figshare.32122714)) and are publicly available as of the data of publication.

Research involving human participants, their data, or biological material

Policy information about studies with human participants or human data. See also policy information about sex, gender (identity/presentation), and sexual orientation and race, ethnicity and racism.

Reporting on sex and gender	This study did not involve human participants, human data, or human biological material.
Reporting on race, ethnicity, or other socially relevant groupings	This study did not involve human participants, human data, or human biological material.
Population characteristics	This study did not involve human participants, human data, or human biological material.
Recruitment	This study did not involve human participants, human data, or human biological material.
Ethics oversight	This study did not involve human participants, human data, or human biological material.

Note that full information on the approval of the study protocol must also be provided in the manuscript.

Field-specific reporting

Please select the one below that is the best fit for your research. If you are not sure, read the appropriate sections before making your selection.

☒ Life sciences ☐ Behavioural & social sciences ☐ Ecological, evolutionary & environmental sciences

For a reference copy of the document with all sections, see nature.com/documents/nr-reporting-summary-flat.pdf

Life sciences study design

All studies must disclose on these points even when the disclosure is negative.

Sample size	Sample sizes were determined based on preliminary observations of variability, ensuring sufficient power to detect meaningful differences.
Data exclusions	Data points considered as outliers (data points affected by technical issues such as staining errors, and values beyond 1.5 times the interquartile range) were removed prior to statistical analysis.
Replication	To assess reproducibility, the experiment was performed on different days, and reproducibility was confirmed.
Randomization	In this paper, rearing conditions, age, and genetic background were standardized. Therefore, it was unnecessary to control for these covariates in the statistical analysis.
Blinding	Investigators were blinded to group allocation during data collection and analysis.

Reporting for specific materials, systems and methods

We require information from authors about some types of materials, experimental systems and methods used in many studies. Here, indicate whether each material, system or method listed is relevant to your study. If you are not sure if a list item applies to your research, read the appropriate section before selecting a response.

Materials & experimental systems

n/a	Involved in the study	
☐	☒	Antibodies
☒	☐	Eukaryotic cell lines
☒	☐	Palaeontology and archaeology
☐	☒	Animals and other organisms
☒	☐	Clinical data
☒	☐	Dual use research of concern
☒	☐	Plants

Methods

n/a	Involved in the study	
☒	☐	ChIP-seq
☒	☐	Flow cytometry
☒	☐	MRI-based neuroimaging

Antibodies

Antibodies used	rat anti-GFP antibody (Nacalai Tesque, #04404-26), rabbit anti-GFP antibody (Invitrogen, #A6455), rabbit anti-Phospho-Myosin Light Chain 2 (Ser19) antibody (CST, #3671), rabbit anti-Zfh1 antibody (R. Lehmann), abbit anti-DsRed antibody (Clontech, # 632496), mouse anti-Gasp antibody (DSHB, #2A12), anti-Mmp1 antibody (DSHB, 1:10 from 1:1:1 cocktail of 3A6B4, 3B8D12 and 5H7B11),

	rabbit anti-Collagen IV antibody (E. Hirooka et al., 2025), Goat anti-rabbit Alexa 488 (Invitrogen, #A11034), Goat anti-rabbit Alexa 546 (Invitrogen, #A11035), Goat anti-rabbit Alexa 647 (Invitrogen, #A21245), Goat anti-rat Alexa 488 (Invitrogen, #A11006), Goat anti-mouse Alexa 546 (Invitrogen, #A11018), Goat anti-mouse Alexa 647 (Invitrogen, #A21237)
Validation	No experimental validation of the primary antibodies was performed in this study.

Animals and other research organisms

Policy information about studies involving animals; ARRIVE guidelines recommended for reporting animal research, and Sex and Gender in Research

Laboratory animals	Drosophila melanogaster
Wild animals	This study did not involve wild animals.
Reporting on sex	To our knowledge, no clear sex-dependent differences in ECM degradation have been reported during Drosophila wing morphogenesis; therefore, sex was not included as a variable in the analysis.
Field-collected samples	This study did not involve samples collected from field.
Ethics oversight	All experiments were conducted in accordance with the guidelines of Nagoya University and were approved by the Instituonal Animal Care and Use Committee (protocol number:560).

Note that full information on the approval of the study protocol must also be provided in the manuscript.

Plants

Seed stocks	This study did not involve plants.
Novel plant genotypes	This study did not involve plants.
Authentication	This study did not involve plants.
